# *catena*-Poly[[(ethane­diol-κ^2^ *O*,*O*′)zinc]-μ-oxalato-κ^4^ *O* ^1^,*O* ^2^:*O* ^1′^,*O* ^2′^]

**DOI:** 10.1107/S1600536812024361

**Published:** 2012-06-02

**Authors:** Zheng-De Tan, Feng-Jiao Tan, Bo Tan, Cheng-Ming Zhang

**Affiliations:** a College of Chemistry and Chemical Engineering, Hunan Institute of Engineering, Xiangtan 411104, People’s Republic of China; b The People’s Hospital of Xiangtan County, Xiangtan 411104, People’s Republic of China

## Abstract

In the title complex, [Zn(C_2_O_4_)(C_2_H_6_O_2_)]_*n*_, the Zn^II^ ion is in a distorted octa­hedral environment formed by two O atoms from an ethyl­ene glycol mol­ecule and four O atoms from two oxalate anions. The oxalate anions link the Zn^II^ ions, forming a zigzag chain along [010]. The zigzag chains are extended into a three-dimensional network by O—H⋯O hydrogen bonds.

## Related literature

For related structures of complexes with oxalates, see: Jin & Lin (2011 ▶); Shen & Lush (2012 ▶).
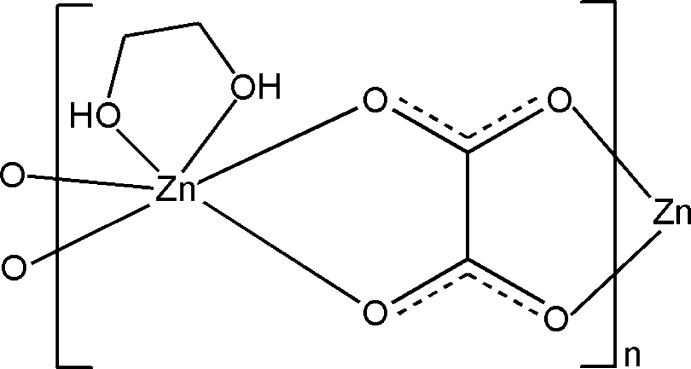


## Experimental

### 

#### Crystal data

[Zn(C_2_O_4_)(C_2_H_6_O_2_)]
*M* *_r_* = 215.46
Orthorhombic, 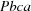

*a* = 7.6411 (15) Å
*b* = 9.3603 (19) Å
*c* = 19.589 (4) Å
*V* = 1401.1 (5) Å^3^
*Z* = 8
Mo *K*α radiation
μ = 3.49 mm^−1^
*T* = 293 K
0.26 × 0.25 × 0.24 mm

#### Data collection

Rigaku SCXmini CCD diffractometer
Absorption correction: multi-scan (*CrystalClear*; Rigaku, 2005 ▶) *T*_min_ = 0.464, *T*_max_ = 0.488
11048 measured reflections
1258 independent reflections
1064 reflections with *I* > 2σ(*I*)
*R*_int_ = 0.068

#### Refinement

*R*[*F* ^2^ > 2σ(*F* ^2^)] = 0.043
*wR*(*F* ^2^) = 0.133
*S* = 0.97
1258 reflections
108 parameters
2 restraints
H atoms treated by a mixture of independent and constrained refinement
Δρ_max_ = 0.41 e Å^−3^
Δρ_min_ = −0.29 e Å^−3^

### 

Data collection: *CrystalClear* (Rigaku, 2005 ▶); cell refinement: *CrystalClear*; data reduction: *CrystalClear*; program(s) used to solve structure: *SHELXS97* (Sheldrick, 2008 ▶); program(s) used to refine structure: *SHELXL97* (Sheldrick, 2008 ▶); molecular graphics: *XP* in *SHELXTL* (Sheldrick, 2008 ▶) and *DIAMOND* (Brandenburg & Putz, 1999 ▶); software used to prepare material for publication: *SHELXTL*.

## Supplementary Material

Crystal structure: contains datablock(s) I, global. DOI: 10.1107/S1600536812024361/hy2552sup1.cif

Structure factors: contains datablock(s) I. DOI: 10.1107/S1600536812024361/hy2552Isup2.hkl

Additional supplementary materials:  crystallographic information; 3D view; checkCIF report

## Figures and Tables

**Table 1:** Hydrogen-bond geometry (Å, °)

*D*—H⋯*A*	*D*—H	H⋯*A*	*D*⋯*A*	*D*—H⋯*A*
O5—H5⋯O1^i^	0.82 (1)	1.88 (2)	2.689 (5)	170 (7)
O6—H6⋯O2^ii^	0.82 (1)	1.91 (2)	2.717 (5)	169 (6)

Symmetry codes: (i) 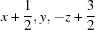; (ii) 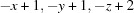.

**Fig. 1.**
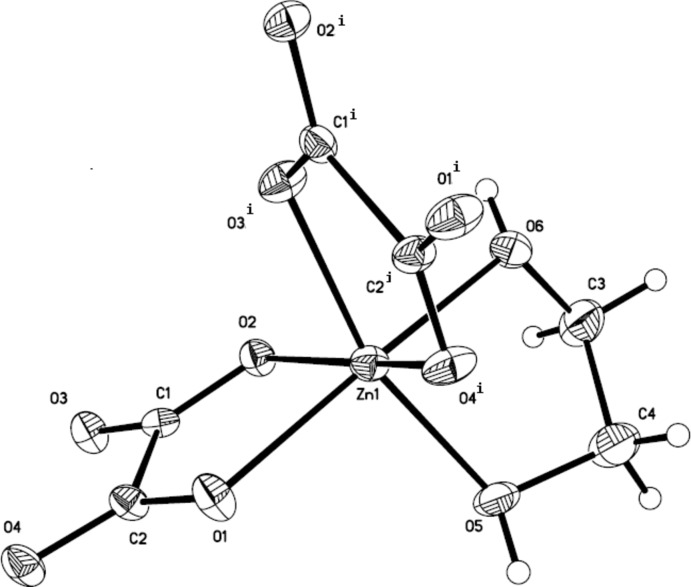
The asymmetric unit of the title compound. Displacement ellipsoids are drawn at the 30% probability level. [Symmetry code: (i) 1/2-x, -1/2+y, z.]

**Fig. 2.**
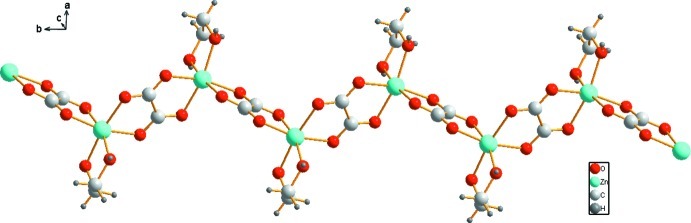
The one-dimensional zigzag chain in the title compound.

**Fig. 3.**
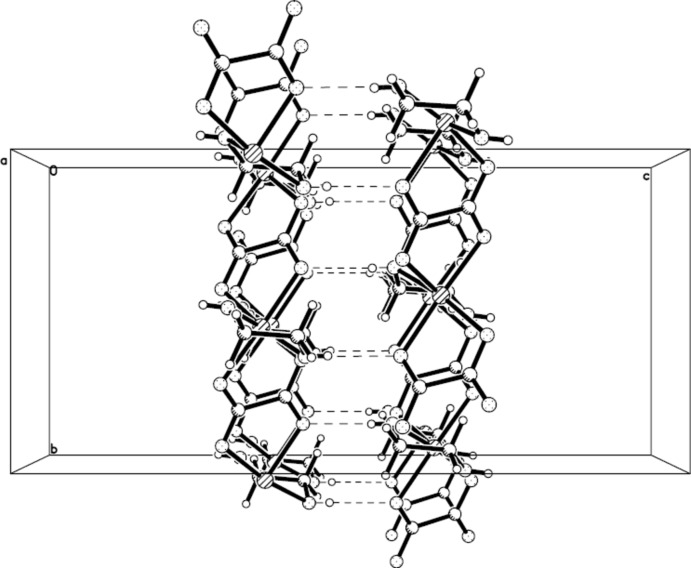
Crystal packing of the title compound. Dashed lines denote hydrogen bonds.

## supplementary crystallographic information

### Comment

Oxalate is a very useful ligand for constructing coordination polymers (Shen & Lush, 2012) and it can be obtained as the degradation of some organic ligands (Jin & Lin, 2011). In this paper, we obtained the oxalate ligand by the oxidation of ethylene glycol *in situ* by solvothermal method. In the title compound, the Zn^II^ ion is in a distorted octahedral environment formed by two O atoms from a chelate ethylene glycol molecule and four O atoms from two different oxalate anions (Fig. 1). The oxalate anions link the Zn^II^ ions, leading to a zigzag chain structure along [0 1 0] (Fig. 2). The zigzag chains are extended into a three-dimensional structure by O—H···O hydrogen bonds (Fig. 3 and Table 1).

### Experimental

A mixture of Zn(NO_3_)_2_.6H_2_O (0.148 g, 0.5 mmol) and concentrated sulfuric acid (0.5 ml) in ethylene glycol (10 ml) was placed in a 23 ml Teflon-lined stainless steel reactor and heated at 383 K for 48 h. After cooling to room temperature over a period of 48 h, colorless crystals suitable for X-ray analysis were obtained.

### Refinement

C-bound H atoms were placed at calculated positions and refined as riding atoms, with C—H = 0.97 Å and with *U*_iso_(H) = 1.2*U*_eq_(C). H atoms on O atoms were located in a difference Fourier map and refined isotropically, with a distance restraint of O—H = 0.82 (1) Å.

### Crystal data

**Table:**

[Zn(C_2_O_4_)(C_2_H_6_O_2_)]	*F*(000) = 864
*M**_r_* = 215.46	*D*_x_ = 2.043 Mg m^−^^3^
Orthorhombic, *P**b**c**a*	Mo *K*α radiation, λ = 0.71073 Å
Hall symbol: -P 2ac 2ab	Cell parameters from 11377 reflections
*a* = 7.6411 (15) Å	θ = 3.0–27.6°
*b* = 9.3603 (19) Å	µ = 3.49 mm^−^^1^
*c* = 19.589 (4) Å	*T* = 293 K
*V* = 1401.1 (5) Å^3^	Block, colorless
*Z* = 8	0.26 × 0.25 × 0.24 mm

### Data collection

**Table:**

Rigaku SCXmini CCD diffractometer	1258 independent reflections
Radiation source: fine-focus sealed tube	1064 reflections with *I* > 2σ(*I*)
Graphite monochromator	*R*_int_ = 0.068
ω scans	θ_max_ = 25.2°, θ_min_ = 3.4°
Absorption correction: multi-scan (*CrystalClear*; Rigaku, 2005)	*h* = −9→9
*T*_min_ = 0.464, *T*_max_ = 0.488	*k* = −11→11
11048 measured reflections	*l* = −23→23

### Refinement

**Table:**

Refinement on *F*^2^	Primary atom site location: structure-invariant direct methods
Least-squares matrix: full	Secondary atom site location: difference Fourier map
*R*[*F*^2^ > 2σ(*F*^2^)] = 0.043	Hydrogen site location: inferred from neighbouring sites
*wR*(*F*^2^) = 0.133	H atoms treated by a mixture of independent and constrained refinement
*S* = 0.97	*w* = 1/[σ^2^(*F*_o_^2^) + (0.1*P*)^2^ + 0.250*P*] where *P* = (*F*_o_^2^ + 2*F*_c_^2^)/3
1258 reflections	(Δ/σ)_max_ = 0.001
108 parameters	Δρ_max_ = 0.41 e Å^−^^3^
2 restraints	Δρ_min_ = −0.29 e Å^−^^3^

### Special details

**Table:**

Geometry. All e.s.d.'s (except the e.s.d. in the dihedral angle between two l.s. planes) are estimated using the full covariance matrix. The cell e.s.d.'s are taken into account individually in the estimation of e.s.d.'s in distances, angles and torsion angles; correlations between e.s.d.'s in cell parameters are only used when they are defined by crystal symmetry. An approximate (isotropic) treatment of cell e.s.d.'s is used for estimating e.s.d.'s involving l.s. planes.
Refinement. Refinement of *F*^2^ against ALL reflections. The weighted *R*-factor *wR* and goodness of fit *S* are based on *F*^2^, conventional *R*-factors *R* are based on *F*, with *F* set to zero for negative *F*^2^. The threshold expression of *F*^2^ > σ(*F*^2^) is used only for calculating *R*-factors(gt) *etc*. and is not relevant to the choice of reflections for refinement. *R*-factors based on *F*^2^ are statistically about twice as large as those based on *F*, and *R*- factors based on ALL data will be even larger.

### Fractional atomic coordinates and isotropic or equivalent isotropic displacement parameters (Å^2^)

**Table:**

	*x*	*y*	*z*	*U*_iso_*/*U*_eq_	
Zn1	0.43052 (8)	0.45088 (6)	0.86776 (3)	0.0252 (3)	
O1	0.2638 (5)	0.5709 (3)	0.80505 (17)	0.0313 (9)	
O2	0.4012 (4)	0.6358 (4)	0.92671 (16)	0.0261 (8)	
O3	0.2619 (5)	0.8446 (4)	0.92532 (17)	0.0307 (9)	
O4	0.1094 (5)	0.7728 (4)	0.80610 (17)	0.0324 (9)	
O5	0.6647 (5)	0.5109 (5)	0.8235 (2)	0.0382 (9)	
O6	0.6152 (5)	0.3630 (5)	0.93473 (19)	0.0360 (9)	
C1	0.3000 (6)	0.7272 (5)	0.9006 (2)	0.0226 (10)	
C2	0.2171 (6)	0.6879 (5)	0.8305 (2)	0.0234 (11)	
C3	0.7851 (8)	0.4251 (8)	0.9253 (3)	0.0510 (18)	
H3A	0.8742	0.3650	0.9457	0.061*	
H3B	0.7902	0.5187	0.9465	0.061*	
C4	0.8139 (9)	0.4373 (8)	0.8507 (3)	0.0516 (18)	
H4A	0.9202	0.4906	0.8414	0.062*	
H4B	0.8245	0.3433	0.8303	0.062*	
H6	0.597 (7)	0.368 (7)	0.9757 (8)	0.045 (18)*	
H5	0.690 (9)	0.520 (8)	0.7831 (11)	0.06 (2)*	

### Atomic displacement parameters (Å^2^)

**Table:**

	*U*^11^	*U*^22^	*U*^33^	*U*^12^	*U*^13^	*U*^23^
Zn1	0.0290 (4)	0.0245 (4)	0.0222 (4)	−0.0007 (2)	0.0003 (2)	0.0003 (2)
O1	0.042 (2)	0.0265 (19)	0.0256 (19)	0.0090 (16)	−0.0116 (16)	−0.0070 (16)
O2	0.0293 (18)	0.0275 (19)	0.0213 (17)	0.0034 (15)	−0.0039 (14)	−0.0025 (15)
O3	0.037 (2)	0.029 (2)	0.0263 (18)	0.0066 (16)	−0.0081 (16)	−0.0075 (16)
O4	0.042 (2)	0.035 (2)	0.0207 (18)	0.0090 (17)	−0.0090 (15)	−0.0024 (17)
O5	0.031 (2)	0.057 (2)	0.026 (2)	−0.0013 (19)	0.0066 (18)	0.0115 (19)
O6	0.037 (2)	0.047 (2)	0.024 (2)	0.0084 (18)	−0.0009 (17)	0.0094 (19)
C1	0.026 (3)	0.023 (2)	0.019 (2)	−0.005 (2)	0.002 (2)	0.001 (2)
C2	0.030 (3)	0.023 (3)	0.018 (2)	0.001 (2)	−0.005 (2)	−0.002 (2)
C3	0.032 (3)	0.086 (5)	0.035 (3)	0.011 (3)	−0.001 (3)	0.009 (3)
C4	0.036 (4)	0.081 (5)	0.037 (3)	0.001 (3)	0.003 (3)	0.011 (3)

### Geometric parameters (Å, º)

**Table:**

Zn1—O5	2.066 (4)	O5—C4	1.434 (7)
Zn1—O4^i^	2.081 (3)	O5—H5	0.82 (1)
Zn1—O2	2.092 (3)	O6—C3	1.434 (8)
Zn1—O6	2.095 (4)	O6—H6	0.82 (1)
Zn1—O1	2.096 (3)	C1—C2	1.557 (6)
Zn1—O3^i^	2.103 (3)	C3—C4	1.482 (8)
O1—C2	1.255 (5)	C3—H3A	0.9700
O2—C1	1.262 (6)	C3—H3B	0.9700
O3—C1	1.236 (6)	C4—H4A	0.9700
O4—C2	1.239 (6)	C4—H4B	0.9700
			
O5—Zn1—O4^i^	95.80 (15)	C3—O6—Zn1	111.7 (3)
O5—Zn1—O2	95.72 (15)	C3—O6—H6	105 (4)
O4^i^—Zn1—O2	165.25 (15)	Zn1—O6—H6	118 (4)
O5—Zn1—O6	77.65 (15)	O3—C1—O2	126.0 (4)
O4^i^—Zn1—O6	98.49 (16)	O3—C1—C2	117.4 (4)
O2—Zn1—O6	92.94 (15)	O2—C1—C2	116.5 (4)
O5—Zn1—O1	97.75 (15)	O4—C2—O1	126.5 (4)
O4^i^—Zn1—O1	90.01 (13)	O4—C2—C1	117.3 (4)
O2—Zn1—O1	79.34 (12)	O1—C2—C1	116.2 (4)
O6—Zn1—O1	170.66 (16)	O6—C3—C4	107.0 (5)
O5—Zn1—O3^i^	163.54 (15)	O6—C3—H3A	110.3
O4^i^—Zn1—O3^i^	80.20 (13)	C4—C3—H3A	110.3
O2—Zn1—O3^i^	91.17 (13)	O6—C3—H3B	110.3
O6—Zn1—O3^i^	87.11 (16)	C4—C3—H3B	110.3
O1—Zn1—O3^i^	98.21 (15)	H3A—C3—H3B	108.6
C2—O1—Zn1	114.1 (3)	O5—C4—C3	106.5 (5)
C1—O2—Zn1	113.8 (3)	O5—C4—H4A	110.4
C1—O3—Zn1^ii^	112.0 (3)	C3—C4—H4A	110.4
C2—O4—Zn1^ii^	112.8 (3)	O5—C4—H4B	110.4
C4—O5—Zn1	113.7 (3)	C3—C4—H4B	110.4
C4—O5—H5	103 (5)	H4A—C4—H4B	108.6
Zn1—O5—H5	130 (5)		

Symmetry codes: (i) −*x*+1/2, *y*−1/2, *z*; (ii) −*x*+1/2, *y*+1/2, *z*.

### Hydrogen-bond geometry (Å, º)

**Table:**

*D*—H···*A*	*D*—H	H···*A*	*D*···*A*	*D*—H···*A*
O5—H5···O1^iii^	0.82 (1)	1.88 (2)	2.689 (5)	170 (7)
O6—H6···O2^iv^	0.82 (1)	1.91 (2)	2.717 (5)	169 (6)

Symmetry codes: (iii) *x*+1/2, *y*, −*z*+3/2; (iv) −*x*+1, −*y*+1, −*z*+2.
